# Increased level of chromosomal damage after irradiation of lymphocytes from *BRCA1* mutation carriers

**DOI:** 10.1038/sj.bjc.6602912

**Published:** 2005-12-13

**Authors:** Z Kote-Jarai, A Salmon, T Mengitsu, M Copeland, A Ardern-Jones, I Locke, S Shanley, B Summersgill, Y-j Lu, J Shipley, R Eeles

**Affiliations:** 1 Translational Cancer Genetics Team, The Institute of Cancer Research, 15 Cotswold Rd, Sutton Surrey SM2 5NG, UK; 2 Sharett Institute of Oncology, Hadassah University Medical Center, Jerusalem 92000, Israel; 3 Royal Marsden NHS Foundation Trust, Fulham Rd, London SW3 6JJ, UK; 4 Molecular Cytogenetics, The Institute of Cancer Research, 15 Cotswold Rd, Sutton Surrey SM2 5NG, UK

**Keywords:** breast cancer, *BRCA1*, chromosomal damage, irradiation, M-FISH

## Abstract

Deleterious mutations in the *BRCA1* gene predispose women to an increased risk of breast and ovarian cancer. Many functional studies have suggested that BRCA1 has a role in DNA damage repair and failure in the DNA damage response pathway often leads to the accumulation of chromosomal aberrations. Here, we have compared normal lymphocytes with those heterozygous for a *BRCA1* mutation. Short-term cultures were irradiated (8Gy) using a high dose rate and subsequently metaphases were analysed by 24-colour chromosome painting (M-FISH). We scored the chromosomal rearrangements in the metaphases from five *BRCA1* mutation carriers and from five noncarrier control samples 6 days after irradiation. A significantly higher level of chromosomal damage was detected in the lymphocytes heterozygous for *BRCA1* mutations compared with normal controls; the average number of aberrations per mitosis was 3.48 compared with 1.62 in controls (*P*=0.0001). This provides new evidence that heterozygous mutation carriers have a different response to DNA damage compared with noncarriers and that *BRCA1* has a role in DNA damage surveillance. Our finding has implications for treatment and screening of *BRCA1* mutation carriers using modalities that involve irradiation.

It is estimated that 5–10% of breast cancer patients develop the disease due to the presence of a highly penetrant breast cancer predisposition gene. A significant proportion of these patients (20–45%) have a mutation in the breast cancer genes, *BRCA1* or *BRCA2* (Peto *et al*, 1999). It has long been debated whether women developing *BRCA*-associated breast cancer have a different response to irradiation. This would be clinically important since, if true, irradiation treatments could lead to more severe acute and late radiotoxicity and an increased carcinogenic risk for these individuals. Hence, the optimum management of *BRCA1* mutation carriers remains unclear (Haffty *et al*, 2002). Elevated chromatid break frequency and various chromosomal abnormalities in cells from individuals with many cancer-prone genetic disorders have been long recognised (reviewed in Eyfjord and Bodvarsdottir, 2005). These abnormalities result from an alteration in chromatin structure, a higher rate of conversion of double-strand breaks (DSB) to chromatid breaks or from deficient DNA repair (Radford, 2004). Following DNA damage, cells have a complex response that may involve cell cycle checkpoint activation, DNA repair mechanisms and programmed cell death (apoptosis). So far, there is evidence that *BRCA1* has a role in all these interdependent events (Kote-Jarai and Eeles, 1999). BRCA1 is a target for phosphorylation by ATM, ATR and Chk2 triggered by DNA damage and is required for cell cycle checkpoint activation (Shiloh, 2003). BRCA1 interacts with the MRE11-Rad50-NBS1 complex (Zhong *et al*, 1999), which is involved in the homologous recombination pathway of DSB DNA repair. A role for BRCA1 in chromatin remodelling and activation of transcription have also been described (Bochar *et al*, 2000).

The phenotypes of cells heterozygous for *BRCA1* mutation have been studied both *in vivo* and *in vitro*. Clinical studies of women with *BRCA1* mutations treated with radiation for breast cancer have not demonstrated increased acute or late toxicity (Pierce, 2005). The *in vitro* studies, however, are suggestive of differences between *BRCA1* heterozygous cells and controls, but no clear evidence as yet has emerged for the impaired efficiency of DNA damage response mechanisms in these cells. Human fibroblasts and lymphoblastoid cells with heterozygous *BRCA1* mutations seem to have heightened radiosensitivity in some assays (Buchholz *et al*, 2002) but not in others (Nieuwenhuis *et al*, 2002). Lymphocytes from *BRCA1/2* carriers have shown increased radiosensitivity as measured by cell survival (Rothfuss *et al*, 2000). Here, we have quantified irradiation-induced chromosome damage in the lymphocytes of *BRCA1* carriers and controls in order to identify a phenotypic effect of *BRCA1* heterozygous mutations.

## METHODS

Individuals heterozygous for *BRCA1* germline mutations were identified from the *BRCA1* and *BRCA2* predictive testing programme in the Institute of Cancer Research/Royal Marsden Foundation NHS Trust, Cancer Genetics Carrier Clinic. Fresh blood samples were collected from five unaffected *BRCA1* heterozygous gene mutation carriers and five healthy age-matched control women with no individual or family history of cancer, and short-term lymphocyte cultures were established. Written informed consent was obtained from all participating individuals prior to inclusion, and the study protocol was approved by the Royal Marsden Locoregional Ethics Committee. Cells were cultured for 24 h and 6 days following irradiation with 8 Gray (Gy) at a high dose rate (0.86 Gy/min) using a Co^60^ source. Metaphase spreads were prepared according to standard methods using 50 *μ*l colcemid for 2 h. Multicolour FISH for karyotyping was performed using the SpectraVision™ Assay system (Abbott Laboratories, Maidenhead, UK). SpectraVision™ probes were hybridised to the chromosome preparations for 48 h, then slides were washed and counterstained with DAPI. Images of metaphase spreads were captured using a Zeiss epifluorescent microscope with a six position filter wheel and were analysed using the Quips SpectraVision™ software (Abbott, as above). An average of 30 metaphases per sample was analysed by an investigator blinded to the genetic status of the cells. A second cytogeneticist (BS), again blinded, independently scored a subset of four samples (two carriers and two controls) to assess interobserver variation. There was a complete concordance between the observations. Chromosomal translocations and breakages were counted per mitosis in *BRCA1* mutation carriers and controls and compared by using the unpaired Student's *t*-test.

## RESULTS

**Figure 1:**
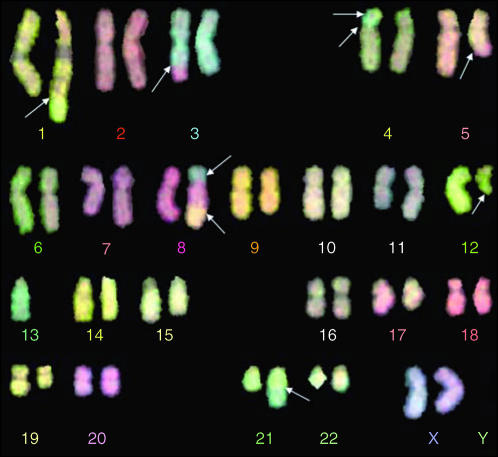
M-FISH analysis of a metaphase spread from a *BRCA1* carrier lymphocyte 6 days after high dose ionizing irradiation (8 Gy total). White arrows show the chromosomal aberrations.

In this study, we evaluated a total of 288 metaphases and chromosomal aberrations were scored per mitosis. At 6 days postirradiation, aberrations were detected. A representative metaphase is shown in Figure 1. Analysis of the average number of aberrations per mitosis revealed an increased level of chromosomal aberrations in heterozygous *BRCA1* mutation carriers compared with controls. In mutation carriers, the average number of chromosomal aberrations was 3.48±0.24 per mitosis, whereas in the control samples, this number was 1.62±0.33 at 6 days post-irradiation. The difference between controls and carriers is highly significant, *P*=0.0001 (Table 1). All chromosomes were equally involved in translocations or breaks when the data were corrected for chromosomal length. No breakage ‘hotspots’ were identified.

At the earlier time point of 24 h post-radiation, based on the analysis of four samples (two carriers, two controls), we did not find a difference between *BRCA1* mutation carriers and controls. The number of average chromosome aberrations/mitosis varied between 0.7 and 2.0 independent of genotype. This suggests that the substantially higher level of chromosomal damage at 6 days reflects the heterozygous cells' lack of ability to recover or apoptose from irradiation-induced damage. This has not been previously described.

Our data indicate that after high dose irradiation, lymphocytes which are heterozygous for a *BRCA1* mutation have inefficient DNA repair and/or apoptotic mechanisms leading to survival of cells with complex chromosomal aberrations. This observation confirms the DNA damage surveillance role of *BRCA1* and could also have an impact on the clinical management of patients carrying a *BRCA1* mutation.

## DISCUSSION

Our data provide evidence that 6 days after high dose irradiation, normal cells (lymphocytes) heterozygous for a *BRCA1* mutation develop a significantly higher level of chromosomal aberrations when compared with controls. Although the 8 Gy dose given here to lymphocytes is higher than that which would be used in a single screening mammogram or fraction of therapeutic radiation, these data emphasise that there is a distinct heterozygous phenotype in normal human cells harbouring a *BRCA1* mutation. It is manifest in the development of twice the number of chromosomal aberrations after irradiation that is only seen after 6 days and is not seen acutely (within 24 h).

It has been shown that gross chromosomal changes are more likely to occur in cancers occurring in individuals with germline mutations in *BRCA1* and *BRCA2* compared with sporadic cancers. CGH analysis has identified a pattern of genetic imbalances that can differentiate familial *BRCA1* tumours from unselected sporadic tumours (van Beers *et al*, 2005). This indicates that some changes might be of functional significance in tumour progression or might represent damage-prone fragile sites.

Recently, it has been shown that BRCA1 is required for common fragile-site stability. Cells lacking BRCA1 show an increased expression of specific common fragile sites (Arlt *et al*, 2004). This provides further evidence that cells lacking BRCA1 are likely to be prone to genomic alterations that can lead to deletion of associated genes and this consequently could promote tumourigenesis.

Our findings might also have some important clinical implications. It raises the possibility that there may be long-term risks of the development of chromosomal instabilities after irradiation of *BRCA*1 mutation carriers.

Even though the 8 Gy dose used in our experimental system exceeded the 2 Gy single fraction size commonly used in a breast cancer radiotherapy regimes, we have to consider that subsequent and repeated exposure to radiation might have a long-term and additive DNA damage effect. Cells with different genetic backgrounds might respond differently to this. We have used lymphocytes, rather than breast epithelial cells, in our study as these are easily collected and the primary aim was to investigate whether a distinct functional heterozygous phenotype for *BRCA1* carriers exists which may lead to the development of a clinically useful assay. However, there is also experimental evidence that ionising radiation induces various molecular changes in breast epithelial cells and that a *BRCA1*-mutated breast cancer cell line shows deficient repair and increased chromosomal aberration (Mamon *et al*, 2003). We have also previously reported finding a differential gene expression profile in normal breast fibroblasts in response to DNA damage between *BRCA1* mutation carriers and controls (Kote-Jarai *et al*, 2004), supporting the hypothesis that response to DNA damage due to irradiation differs in *BRCA1* mutation carriers. Consequently, we suggest that clinical follow-up of the screening and treatment of individuals harbouring *BRCA1* mutations is imperative. Studies are urgently needed to elucidate the applicability of these data to the clinical use of irradiation in screening and treatment for the management of *BRCA1* gene mutation carriers. Concerns about the potential mutagenicity of ionising radiation, both radiotherapy and surveillance mammography in this group, have already driven the search for alternative tools such as breast magnetic resonance imaging in the UK MARIBS study (The MARIBS Advisory Group, 2005). Based on cohort studies of long-term mortality and risk estimation models for the induction of secondary cancers following radiotherapy at least 15–20 years follow-up will be required to fully assess whether there is an increased long-term treatment induced cancer risk (Dasu *et al*, 2005; Darby *et al*, 2005) and subtle late toxicity from irradiation in *BRCA1* mutation carriers.

## Figures and Tables

**Table 1:** Average number of chromosomal aberrations per metaphase in *BRCA1* mutation carriers and controls

**Sample no.**	***BRCA1* Genotype**	**Aberration/metaphase**	**Sample no.**	***BRCA1* Genotype**	**Aberration/metaphase**	
1	187_188delAG	3.80	6	Wild type	2.20	
2	2313G>T	3.67	7	Wild type	1.60	
3	Exon 13 duplication	3.36	8	Wild type	1.39	
4	4184_4187delTCAA	3.37	9	Wild type	1.55	
5	1014delGT	3.17	10	Wild type	1.40	
						
Average no of aberration/metaphase		3.48±0.24			1.62±0.33	*P*=0.0001
